# PReS-FINAL-2064: Effect of Goloimumab a new anti-TNF, in patients with the diagnosis of juvenile idiopathic arthritis

**DOI:** 10.1186/1546-0096-11-S2-P76

**Published:** 2013-12-05

**Authors:** A Kienast, I Foeldvari

**Affiliations:** 1 Hamburger Zentrum für Kinder-und Jugendrheumatologie, Hamburg, Germany

## Introduction

Golimumab is a fully human monoclonal antibody targeting tumor necrosis factor-alpha (TNF-α), which plays an important role in the pathogenesis of juvenile idiopathic arthritis. Golimumab was approved for the treatment of rheumatoid arthritis, psoriatic arthritis and ankylosing spondylitis.

## Objectives

To assess the effectivity and safety of Gololimumab in juvenile idiopathic arthritis.

## Methods

We analysed retrospectively the data of all our patients who have been treated with Golimumab (Simponi^®^) every 28 days sub cutaneously.

## Results

18 patients with the diagnosis of juvenile idiopathic arthritis (15 with juvenile enthesitis associated arthritis, 2 with juvenile psoriatic arthritis and 1 with juvenile idiopathic polyarticular arthritis), for a mean time of 13.7 months per patient (4-26 months). 16 patients have been treated with other biologic agents before. In 13 patients Golimumab was started because of disease progression, in 3 because of intolerance to other biologic agents and in 2 because they described severe phobia of injections.Mean number of painful, swollen and limited joints were before and at the end of the treatment period after 13.7 months (4.5/2.39; 1.78/0.94; 2.72/2.83). The mean value at baseline after the mean follow up were of the physician global assessment score (2.03/0.84), mean erythrocyte sedimentation rate (8.72 mm/7.41 mm) and mean c-reactive protein (4.36 mg/l/3.97 mg/l). 13 patients developed side effects, one patient developed a severe adverse event (appendicitis with consecutive appendectomy). None of the patients was the drug discontinued because of the side effects.

## Conclusion

According to this preliminary study in patients with JIA golimumab seems to be a safe and effective treatment. The data of the prospective study is pending.

## Disclosure of interest

None declared.

